# Ongoing COVID-19 Pandemic Effects on Admissions and In-Hospital Outcomes in Patients With ST-Elevation Myocardial Infarction (STEMI): An Albanian Observational Study

**DOI:** 10.7759/cureus.26813

**Published:** 2022-07-13

**Authors:** Leonard Simoni, Ilir Alimehmeti, Astrit Ceka, Ermir A Tafaj, Mirald Gina, Aldo Panariti, Fatjon Xhafaj, Alban Dibra, Artan Goda

**Affiliations:** 1 Cardiovascular Disease, University Hospital Center "Mother Teresa", Tirana, ALB; 2 Health Commission, Academy of Sciences of Albania, Tirana, ALB; 3 Department of Family and Occupational Health, Faculty of Medicine, University of Medicine, Tirana, ALB; 4 Cardiovascular Disease, University Hospital Center Mother Teresa, Tirana, ALB; 5 Cardiovascular Disease, University Hospital Center 'Mother Teresa", Tirana, ALB

**Keywords:** coronary invasive procedures, ongoing covid-19 pandemic, mortality, hospital admissions, st-elevation myocardial infarction

## Abstract

Background

Multiple studies conducted worldwide and in Albania documented an important reduction of acute ST-elevation myocardial infarction (STEMI) admissions during the Coronavirus Disease 19 (COVID-19) pandemic. There are few studies regarding STEMI admissions and outcomes during the ongoing pandemic after the initial lockdown. We aimed to study STEMI admissions and in-hospital outcomes after the COVID-19 lockdown period.

Methods

A retrospective single-center study was conducted, collecting data for all consecutive STEMI admissions from March 9th, (the first COVID-19 case) until April 30^th^, the corresponding period of 2020 total lockdown, for years 2019 and 2021. The control period was considered the year 2019 [pre-pandemic (PP)] and the study period was in 2021 [ongoing pandemic (OP)]. The incidence rate ratio (IRR) 95% confidence interval (CI) was used to compare all-STEMI admissions, invasive procedures, and risk ratio (RR) 95% CI to compare the mortality and complications rate between the study and control period.

Results

The study included 217 STEMI patients admitted in 2019, and 234 patients during the 2021 period. The overall-STEMI admissions IRR is in a similar range during the 2021 OP compared to the 2019 PP period IRR=1.07 (95%CI 0.90-1.28). Similar invasive procedures were observed during OP compared to PP period, respectively for coronary-angiography IRR= 1.07; (0.87-1.31), for all-PCI [1.12 (0.92-1.35)], and primary percutaneous coronary interventions (PCI) [1.09 (0.89-1.34)]. The STEMI death rate during OP compared to PP period was similar (7.3 vs. 7.4%), RR=1.01 (0.53-1.96), and a non-significant lower primary-PCI-death rate (4.0 vs 4.8%), RR= 0.83 (0.30-2.3)].

Conclusions

After the initial reduction of admissions and invasive procedures in STEMI patients during the 2020 lockdown period and the increase of all-STEMI mortality, the number of hospitalizations, invasive procedures, and mortality returned to a similar range during OP compared to the PP period despite a highly incident ongoing COVID-19 pandemic.

## Introduction

During the first wave of COVID-19 pandemic outbreak, an important drop in all Acute Coronary Syndromes (ACS) [1-4] and particularly in ST-elevation myocardial infarction (STEMI) admissions [5-8] and related invasive procedures [9,10] were reported globally. The first case of COVID-19 in Albania was declared on the 9th of March 2020 [11] followed by a cascade of restrictive measures and the installation of a total lockdown on the 15th of March 2020 [12]. The first release measures began at the end of April and continued through May, restoring the “normal” life [13]. We previously documented a significant reduction of all-ACS (-41.6%) [14] and STEMI admissions (-28%) [15], and also an increase in mortality for all-ACS with a risk ratio (RR) = 2.2 [(1.20-3.89); p=0.01] and STEMI [RR =1.91 (1.039-3.52) p=0.037] during the lockdown in 2020 compared to the pre-pandemic (PP) period.

The installation of total lockdown measures was not undertaken anymore in Albania, even though during the ongoing pandemic there were other high incidence COVID-19 waves throughout October-November 2020 and January - March 2021 [16]. During the entire year of the ongoing pandemic, the population was called to physical distance and the wearing of masks, without impeding normal activities and work. By the end of April 2020, the incidence of active cases in Albania was 6.71/ 100000 inhabitants. The peak of documented active cases during the years 2020-2021 was on March 5th 2021 by 1278.36 /100000 inhabitants demonstrating that the COVID-19 pandemic severely hit the country by that time [16]. Throughout the ongoing pandemic, many countries undertook many other restrictive measures, and closures and also installed other periods of total lockdown with various effects on hospitalizations and outcomes of patients with ACS [(STEMI and Non-ST-elevation acute coronary syndromes (NSTEACS)] [17-24]. Based on our first report for STEMI admissions and outcomes during the lockdown COVID-19 period [15], our purpose was to investigate the admissions and in-hospital outcomes during the same timeframe throughout the ongoing pandemic period in 2021.

## Materials and methods

A retrospective, observational study in the largest tertiary university hospital and the unique public primary percutaneous coronary interventions (PCI) center in Tirana, Albania was conducted. All consecutive patients admitted with STEMI were included. STEMI was diagnosed using the fourth universal definition of myocardial infarction criteria [25]. We presented the study as an abstract oral presentation during the rapid-fire session at the ESC Preventive Cardiology 2022 held on 7-9 April 2022. This study is a continuation of the study previously published [15] regarding the impact of the COVID-19 pandemic on hospitalization and in-hospital outcomes of patients with STEMI. The methodology of the study is already published [15] and will be presented shortly.

The information for all admissions from March 9th, (the first COVID-19 case) until April 30th, the corresponding period of 2020 total lockdown, for the years 2019 and 2021 was collected. The control was considered during the correspondent PP period of the year 2019 and the study was considered during the year 2021, the ongoing pandemic (OP) period. The data of study variables were gathered from medical and procedural files including age, gender, cardiovascular risk factors, comorbidities, angiographic, PCI, and coronary artery bypass grafting (CABG) findings. Angiographic and procedural findings were presented including the number of affected coronary vessels, percutaneous coronary interventions (PCI), and recommended and performed CABG.

Admission rates and in-hospital outcomes analysis

STEMI admissions and procedures and weekly admissions/ procedures of OP and PP are compared. The rate ratio of STEMI hospitalizations/procedures between the OP and the PP is shown as incidence rate ratios (IRR), which were calculated by comparing the incidence ratios of hospitalizations/procedures for STEMI per week for each period. IRR is presented with 95% confidence intervals (95% CI).

The STEMI mortality and cardiogenic shock rate ratio between the OP and PP period are presented in the RR with 95% CIs. Other in-hospital outcomes were analyzed between two periods including the time from symptom onset to intensive care unit (ICU), ICU- sheath insertion time, cardiac troponin I (cTnI) on admission (normal values 0.00-1.00 ng/mL, and left ventricular ejection fraction (LVEF) (at discharge) and hospital length of stay.

Statistical analysis

The Statistical Package for the Social Sciences (SPSS) released in 2012 (IBM Corp, SPSS Statistics for Windows, Version 21.0, Armonk, NY) was used to perform the statistical analysis. T-tests were used to compare the continuous variables and presented as mean ± SD, meanwhile, the chi-squared (χ2) test was used to compare discrete and categoric data, shown as numbers, percentages, and RR with 95% CI. The Poisson regression (STEMI admissions per week model) was used to determine the IRR for admissions, and procedures, between the ongoing pandemic and the pre-pandemic periods. IRR between the groups is presented with a 95% Confidence Interval (95%CI). The two-sided p-value of < 0.05 was considered statistically significant.

## Results

Patients basal characteristics

**Figure 1:**
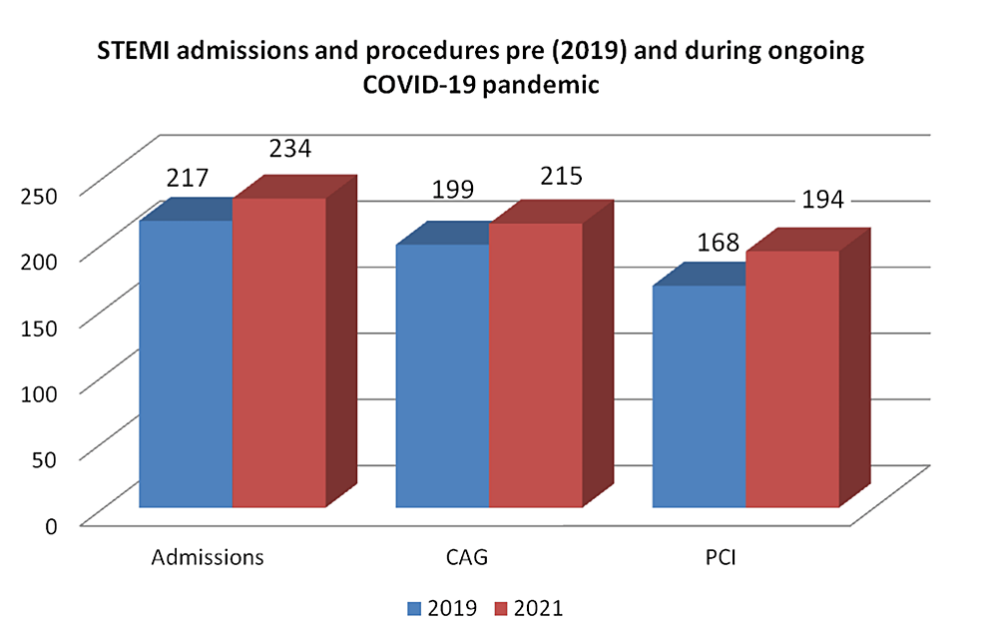
ST‐segment elevation myocardial infarction (STEMI) admissions, coronary-angiography (CAG), and percutaneous coronary intervention (PCI), during PP and OP periods Pre-pandemic (PP) period is shown in blue bars and the ongoing pandemic (OP) period in red bars.

During both PP and OP periods 451 patients were hospitalized with the diagnosis of STEMI, 217 patients (48.1 %) were admitted during the PP period, and 234 patients (51.9%) during the OP period. Table 1 and Figure 1 show the baseline data. No differences between OP and PP patients in baseline characteristics including gender, age, cardiovascular risk factors (arterial hypertension, dyslipidemia, diabetes mellitus, and smoking), and comorbidities were found. No significant differences were also found between OP and PP period in the percentage of performed coronary angiography (91.9% vs. 91.7%), but there is a trend of a higher percentage of performed PCI (82.9% vs 77.4%; p=0.179), and primary PCI (PPCI) (75.2 vs. 67.3% p=0.079) during the OP period. Patients during the OP period presented more vessel or LM disease 43.7 vs. 31.2% p=0.011 (Table 1 and Figure 1). The left anterior descending artery vessel (LAD) was the most affected and treated with PCI in both groups, but no differences between groups were found. Also, no significant differences in the recommended and performed CABG percentage between periods were found (Table 1).

**Table 1:** Baseline demographic, clinical, and angiographic characteristics MI: myocardial infarction, CAD: coronary artery disease, CMP: cardiomyopathy, LM: left main, PCI: percutaneous coronary intervention, PPCI: primary percutaneous coronary intervention, LAD: left anterior descending, LCX: left circumflex, RCA: right coronary artery, CABG: coronary artery bypass grafting. *To determine statistical significance for the comparison regarding each one of demographic characteristics, angiographic and procedure-related variables were summarized using mean ± SD for continuous variables compared using t-tests and frequency and percentage for categorical variables compared using chi-squared (χ2) tests

Variables	Control period (2019) 217 pts n (%)	Ongoing pandemic (2021) 234 pts n(%)	P value
Male	153 (73%)	175 (74.8%)	0.361
Age, yrs (SD)	66.56 (11.46)	65.48 (11.58)	0.283
Diabetes Mellitus	104 (47.9%)	106 (45.3%)	0.642
Arterial hypertension	189 (87.1%)	201 (85.9%)	0.815
Dyslipidema	119 (54.8%)	124 (53.0%)	0.903
Smoking	88 (40.6%)	87 (37.2%)	0.623
Previous MI	29 (13.4%)	35 (14.9%)	0.786
Previous CAD	27 (12.4%)	32 (13.7%)	0.837
Dilated CMP	10 (4.6%)	12 (5.1%)	0.934
Impaired renal function	31 (14.3%)	34 (14.5%)	0.976
Previous stroke	12 (5.5%)	15 (6.4%)	0.885
Coronary Angiography	199 (91.7%)	215 (91.9%)	0.946
1 vessel CAD (% angiography)	53 (26.6%)	58 (27.0%)	0.954
2 vessel CAD (% angiography)	80 (40.2%)	63 (29.3%)	0.026
3 vessel CAD/LM (% angiography)	62 (31.2%)	94 (43.7%)	0.011
No critical stenoses	3 (1.5%)	1 9 (0.4%)	0.184
PCI (% all)	168 (77.4%)	194 (82.9%)	0.179
PPCI (n, %)	146 (67.3%)	176 (75.2%)	0.079
PCI of LAD (% of PCI)	110 (65.5%)	119 (61.3%)	0.785
PCI of LCX (% of PCI)	41 (24.4%)	64 (33.0%)	0.210
PCI of RCA (% of PCI)	80 (47.6%)	87 (44.8%)	0.723
CABG recommended (% angiography)	30 (13.8%)	25 (10.7%)	0.382
CABG performed (% angiography)	5 (2.3%)	13 (5.6%)	0.128
CAD with medical treatment	6 (2.7%)	10 (4.3%)	0.541

Admission and procedural findings

**Figure 2:**
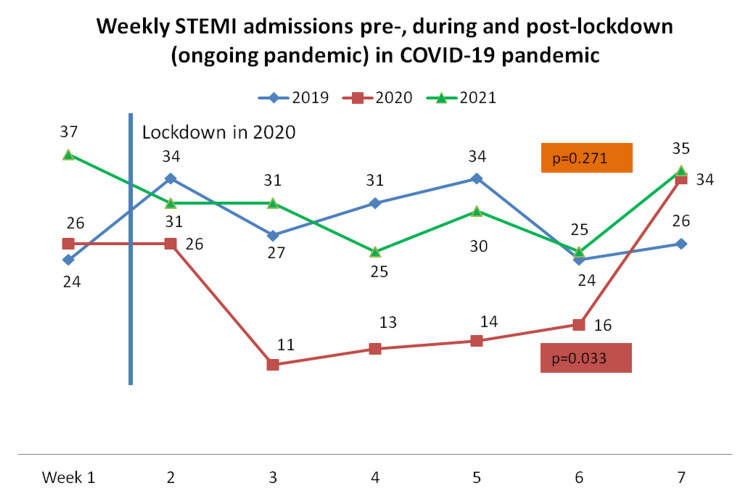
The weekly number of STEMI admissions. Pre-lockdown (blue line), during lockdown (red line), and post-lockdown/ongoing pandemic (green line). This shows an important reduction in STEMI admissions during the lockdown period in 2020 (mainly during the third and fourth week) when compared to the PP period (2019). No significant differences between the OP (2021) and the PP period (2019).

A slight nonsignificant increase of STEMI admissions by 7% was observed during the OP compared with the PP period representing a weekly IRR =1.07 (0.90-1.28) p=0.271, contrary to our previous result for the lockdown period during the pandemic outbreak in 2020 compared to the pre-pandemic period, where a decrease of STEMI admissions by 28.1%, representing an IRR 0.719 (0.56-0.94); p=0.033 (Table 2 and Figure 2). Similar number of patients underwent coronary angiography with an IRR = 1.07 (0.87-1.31) p=0.264, PCI with an IRR = 1.12 (0.92-1.35) p=0.121, and PPCI with an IRR= 1.09 (0.89-1.34) p=0.143 during both periods (Table 2). Figure 2 shows the weekly number of STEMI admissions [pre- (blue line), during (red line), and post-lockdown OP (green line) of COVID-19 pandemic] shows an important reduction in STEMI admissions during the lockdown period (mainly during 3rd and 4th week) compared to the pre-pandemic period 2019. No significant differences between OP (2021) and PP (2019).

**Table 2:** Admissions presentation, and correspondent weekly incidence rate ratio between the OP and the PP periods STEMI: ST‐segment elevation myocardial infarction, PCI: percutaneous coronary intervention, PPCI: primary percutaneous coronary intervention, PP: pre-pandemic, OP: ongoing pandemic. The †Weekly incidence rate ratio for STEMI from the analyses of seven weeks during the OP and the PP period, expressed in incidence rate ratio (IRR) (95%CI). *Poisson regression (STEMI admissions/procedures per week model) was used to calculate IRR for each event between the OP period and the PP period

Admission presentation and procedures	COVID‐19 /ongoing pandemic (2021) n (%)	Pre-pandemic (2019) n(%)	Incidence Rate Ratio (95% CI)†	P‐value*
STEMI (post/pre)	234	217	1.07 (0.90-1.28)	0.271
Coronary angiography	215 (91.9%)	199 (91.7%)	1.07 (0.87-1.31)	0.264
PCI	194 (82.9%)	168 (72.4%)	1.12 (0.92-1.35)	0.121
PPCI	176 (75.2%)	146 (67.3%)	1.09 (0.89-1.34)	0.143

In-hospital outcomes

Similar overall in-hospital STEMI mortality rates were found between the OP and PP periods 7.3% versus 7.4% with a RR of 1.01 (0.53-1.96) p=0.965. Similar in-hospital mortality rates were found also in patients with STEMI undergoing primary PCI 4.0% versus 4.8%, with RR=0.83 (0.30-2.31) p=0.72. Also similar rates were found for cardiogenic shock (CSH) between OP and PP periods 12.8% versus 12.4%, RR=1.03 (0.63-1.67) p=0.904 (Tables 3 and 4).

**Table 3:** In-hospital outcomes PPCI: primary percutaneous coronary intervention, CSH: cardiogenic shock, ICU: intensive care unit, CtnI: cardiac troponin I *To determine statistical significance for the comparison regarding each one of the in-hospital outcomes, frequency and percentage for categorical variables were compared using chi-squared (χ2) tests, and mean with Standard deviation (SD) for continuous variables, compared using T-tests.

Variables	Control period 217 pts (n%)	COVID (post-lockdown 2021) 234 pts (n %)	P value*
Death (% pts)	16(7.4%)	17 (7.3%)	0.965
PPCI Death n (%PPCI)	7(4.8%)	7 (4.0%)	0.933
CSH (% pts)	27 (12.4%)	30 (12.8%)	0.945
Symptom onset-ICU time (SD)	438.73 (385)	401.2 (331.5)	0.311
ICU- sheath time (SD)	57.2±33.2	49.2 (25.6)	0.032
cTn I (SD)	21.96 (49.9)	23.2 (50.2)	0.453
Ejection Fraction (SD)	44.90 (8.9)	45.2 (8.2)	0. 325
Length of stay (SD)	5.98 (3.2)	4.35 (1.1)	< 0.001

Comparable findings were found also regarding the symptom onset to ICU time (401.2±331.5 versus 438.7±385 minutes p = 0.311), first cardiac Troponin I (20.6 ±46.2 versus 21.96 ±49.9 ng/ml p=0.853) and left ventricle ejection fraction (LVEF) on demission (45.2±8.2 vs. 44.9±8.90; p=0.325 among patients admitted during the ongoing compared with the pre-pandemic period. Meanwhile, ICU - sheath insertion time, and the hospital length of stay were shorter during the ongoing pandemic period respectively (49.2±25.6 versus 57.2±33.2; P=0.032) and (4.35±1.1 vs 5.98±3.17 p<0.001).

**Table 4:** Major Complications Risk Ratios in STEMI patients PPCI: primary percutaneous coronary intervention, CSH: cardiogenic shock. †Risk Ratio (RR) for death, PPCI death, and cardiogenic shock STEMI was obtained from the comparison of event rate (death/ CSH) between ongoing COVID-19 and control period and expressed in RR and 95%CI.

Complications	COVID‐19 ongoing (2021) (n%)	Pre-pandemic (2019) n(%)	Risk Ratio (RR)(95% CI)†	P‐value
Death n (%)	17 (7.3%)	16 (7.4%)	1.01 (0.53-1.96).	0.965
PPCI Death n (% PPCI)	7 (4.0%)	7 (4.8%)	0.83(0.30-2.31)	0.721
CSH n (%)	30 (12.8%)	27 (12.4%)	1.03 (0.63-1.67)	0.904

## Discussion

In the present study, we investigated the admissions for STEMI and in-hospital outcomes during the ongoing pandemic in 2021, in comparison with the pre-pandemic period in 2019. We observed that during the ongoing pandemic in 2021, a) the admissions of patients with STEMI, related invasive procedures, coronary angiographies, PCI, and PPCI were comparable with the pre-pandemic period in 2019; b) similar times of hospital presentation from the symptom onset; and c) comparable mortality rates with the pre-pandemic period in 2019.

During the COVID-19 pandemic outbreak in 2020 congruent to many other studies [5-8] our group previously documented an important decrease in admission for STEMI (-28.1), related invasive procedures, and an increase in hospital presentation time [15]. We documented also an important increase by quasi 2 fold in STEMI mortality rates (RR = 1.91 (1.04-3.52); p= 0.037 [15]. A systematic review and meta-analysis [26] of the impact of the SARS-CoV-2 outbreak on the presentation and prognosis of patients with acute myocardial Infarction; including results of 61 studies published throughout the globe, also confirmed the reduction of admissions by 24% (IRR 0.76 [0.67-0.85]) with an impact on early survival (OR = 1.33[1.18- 1.51] in-hospital mortality p=0.02). Also in another meta-analysis conducted by Pourasghari H. et al. [27], a 29% reduction of STEMI was found during the early pandemic period compared to the pre-pandemic period (OR = 0.71; p < 0.001). Meanwhile the STEMI mortality increased by 15% but not statistically significant (OR = 1.15; p = 0.035).

After presenting the initial worldwide abovementioned situation, and with the ongoing more severe pandemic mainly during the last months of 2020 and the first months of 2021, the continuing effect on admissions and mortality from STEMI differs from country to country. Congruent to our findings were observed also in many other countries where the numbers remained “normal” after the early pandemic lockdown period despite ongoing COVID-19. In Denmark [17] the data of the second lockdown( December '20 - January 21) showed a nonsignificant reduction of ischemic heart disease compared with the pre-pandemic period (IRR=0.93 [0.84-1.02]). In Northern Carolina [18], in a study conducted throughout the entire year 2020, after the initial AMI admission reduction, a “normalization” was observed in May and remained stable during the rest of the year. The authors explain the results with the changing patients' attitudes later on during the pandemic or the adaptation and the reassures of the health care system to deal with the pandemics. Also in Germany Brunner S., et al. [19] observed a ‘normalization” of numbers after the initial lockdown despite the ongoing COVID-19 pandemic. The authors suggest that the initial reduction is mainly lockdown-dependent. The release of measures was associated also with the “normalization” of people’s health behavior. Mahli et al. [20] in Canada studied STEMI care across multiple waves of COVID1-19 during 2020 observing similar incidences of STEMI during and before the pandemic. 

On the other side in other countries continuing reduction of STEMI admissions is observed especially related to COVID-19 waves and lockdown periods. One of the first reports come from the UK [21] which observed similar reductions in AMI hospitalizations during the first lockdown from March 23d, 2020 (-32%) and the second one during November 2020 (-34%). The main factor that the authors highlight is the fear of medical structures related to the increase in the number of COVID-19 cases during the waves. In Israel [22], the greatest reduction was observed during the third lockdown (December 2020 to February 2021) by 24% and the post-third lockdown (mid-march) week of 2021 by 22% of STEMI admissions. Reductions in MI were observed also in France in a nationwide study [23] during the second lockdown (weeks 44-50 of 2020) by 8%, even though was smaller than the first lockdown (-24%) (weeks 12-19 of 2020) but without changes in mortality rates.

We previously [15] explained the reduction of STEMI during the outbreak of COVID-19 pandemic lockdown (March- April 2020) with different factors such as a) the fear of catching the virus in medical structures led to a refusal or postponement of medical care; b) the call of STAY AT HOME from the authorities, public and social media information directed almost toward COVID-19 disease, and all restrictive measures might have negatively impacted the seeking of medical care from the population; and c) the direct effect of the lack of physical activity and physical triggers for ischemic heart disease.

In Albania from mid-May began the release of restrictive measures taken from the beginning of the pandemic outbreak bringing the population to a “new normalization” of life. We have to highlight that the lockdown and many other restrictive measures especially of movement inside Albania were not repeated anymore, even though of higher incidence of COVID-19 during the last months of 2020 and the first one of 2021. This “new normalization” probably may have impacted the population to be fearless in seeking health care and not neglecting cardiac visits and consultations. The role of repeated lockdowns and the related negative effect on ACS admissions is suggested also by Katsouras Ch. et al. in their publication [24]. The similar reduction in ACS admissions during the second wave with the first wave of COVID-19 is explained by the possible detrimental indirect effect of lockdowns on cardiovascular diseases in the general population.

Albania through the months changed its approach toward COVID-19 infections. During the first months of the pandemic, the population in every case of dubious or possible COVID-19 was orientated to be directed to national emergency services, which initially might have overwhelmed the services influencing the STEMI hospitalization and transportation toward the University Hospital Center in Tirana. During the second half of 2020, the population was called to contact the family doctors and then if necessary the emergency services. The population was called also not to refuse medical care in general and especially if they have cardiac symptoms. Public information and guarantees given by the health system were at a higher level during the second half of 2020, which probably may have influenced the increase in population belief toward medical structures. The role of public media in better information, and creating greater security for the population is shown also in many other studies. Derks. L et al. [28] documented an increase in acute coronary syndromes admissions in the Netherlands after calls in public media from healthcare professionals encouraging individuals not to postpone medical visits. Same situations. The effects of public calls and media publicity campaigns on the increase in hospital admissions and visits were noted also in England [9].

On the other side, the return to normal work, free of movements, and sports activities mainly in open arias, increasing the physical triggers for ischemic heart disease might have impacted the reverse of admissions for acute myocardial syndromes and especially for myocardial infarction.

Another important factor that may have impacted the reverse of STEMI admissions during 2021 might have been the COVID-19 infection or vaccination for SARS-COV2 per se. We don’t have documented patients that had suffered already the infection, and this is one of the limitations of our study. As for viruses, also SARS-COV2 might increase the risk for ACS associated with worse outcomes through various mechanisms; pro-thrombotic status, inflammatory and immune hyperreactivity leading to destabilization of atherosclerotic plaque, endothelial damage, and other possible mechanisms [29]. A recent systematic review and meta-analysis observed higher in-hospital mortality in the group of patients with concomitant STEMI and SARS-COV-2 compared to those without infection (OR 5.24 [3.63-7.65]) [30].

In our investigation, we documented a ‘normalization’ of the hospitalizations and related invasive procedures during the ongoing COVID-19 pandemic in 2021 compared to the pre-pandemic period in 2019, after an important drop during the COVID-19 pandemic outbreak in 2020. We also documented a ‘normalization’ of mortality during 2021 which turned in a similar range to 2019, after a significant increase during the lockdown period in 2020. In our belief, the changes in population behavior toward the entire pandemic, changes in the management of the COVID-19 pandemic, and the public information in seeking medical care whenever needed are the major reasons for the ‘normalization’ of STEMI hospitalizations and outcomes.

Strength and limitations

Our study is a continuation of the investigation published previously in February 2022 in “The Anatolian Journal of Cardiology”, which compared the lockdown pandemic period with the pre-pandemic period, meanwhile in the present study were included all consecutive patients admitted with STEMI in our center during the ongoing highly incident COVID-19 pandemic in 2021 compared with the same pre-pandemic period, looking inside the tabloid of possible changes of the situation in admissions and outcomes in patients with STEMI.

The patients diagnosed concomitantly with STEMI and COVID-19 were not included because these patients were hospitalized in dedicated COVID hospitals, without having the possibility to investigate the direct impact of COVID-19 on the outcomes. In our study, we did not collect the data of patients that had suffered already the COVID-19 infection or had been vaccinated for SARS-COV2, not knowing if there is any correlation with the ‘normalization of the STEMI situation”. Also, the retrospective collection of the data is another limitation of the study.

## Conclusions

After the initial reduction of admissions and invasive procedures in STEMI patients during the 2020 lockdown period and the increase of all-STEMI mortality, the number of admissions and the mortality returned to a similar range during 2021 despite a highly prevalent ongoing COVID-19 pandemic. Better health structure approach toward the entire pandemic, better public information, changes in the population behavior during the pandemic, and the return to “normal” activities might have impacted the “normalization” of the admissions and outcomes of patients with STEMI.
